# Across the Indian Ocean: A remarkable example of trans-oceanic dispersal in an austral mygalomorph spider

**DOI:** 10.1371/journal.pone.0180139

**Published:** 2017-08-02

**Authors:** Sophie E. Harrison, Mark S. Harvey, Steve J. B. Cooper, Andrew D. Austin, Michael G. Rix

**Affiliations:** 1 Australian Centre for Evolutionary Biology and Biodiversity, School of Biological Sciences, The University of Adelaide, Adelaide, SA, Australia; 2 Department of Terrestrial Zoology, Western Australian Museum, Welshpool DC, WA, Australia; 3 School of Biology, The University of Western Australia, Crawley, WA, Australia; 4 School of Natural Sciences, Edith Cowan University, Joondalup, WA, Australia; 5 Evolutionary Biology Unit, South Australian Museum, North Terrace, Adelaide, SA, Australia; 6 Biodiversity and Geosciences Program, Queensland Museum, South Brisbane, QLD, Australia; Scientific Research Centre of the Slovenian Academy of Sciences and Art, SLOVENIA

## Abstract

The Migidae are a family of austral trapdoor spiders known to show a highly restricted and disjunct distribution pattern. Here, we aim to investigate the phylogeny and historical biogeography of the group, which was previously thought to be vicariant in origin, and examine the biogeographic origins of the genus *Moggridgea* using a dated multi-gene phylogeny. *Moggridgea* specimens were sampled from southern Australia and Africa, and *Bertmainus* was sampled from Western Australia. Sanger sequencing methods were used to generate a robust six marker molecular dataset consisting of the nuclear genes *18S* rRNA, *28S* rRNA, *ITS* rRNA, *XPNPEP3* and *H3* and the mitochondrial gene *COI*. Bayesian and Maximum Likelihood methods were used to analyse the dataset, and the key dispersal nodes were dated using BEAST. Based on our data, we demonstrate that *Moggridgea rainbowi* from Kangaroo Island, Australia is a valid member of the otherwise African genus *Moggridgea*. Molecular clock dating analyses show that the inter-specific divergence of *M*. *rainbowi* from African congeners is between 2.27–16.02 million years ago (Mya). This divergence date significantly post-dates the separation of Africa from Gondwana (95 Mya) and therefore does not support a vicariant origin for Australian *Moggridgea*. It also pre-dates human colonisation of Kangaroo Island, a result which is further supported by the intra-specific divergence date of 1.10–6.39 Mya between separate populations on Kangaroo Island. These analyses provide strong support for the hypothesis that *Moggridgea* colonised Australia via long-distance trans-Indian Ocean dispersal, representing the first such documented case in a mygalomorph spider.

## Introduction

The historical view of the biogeographical history of the Southern Hemisphere postulated that the terrestrial biota had largely vicariant origins [1], and that dispersal played a relatively limited role in taxa with southern-temperate or ‘Gondwanan’ ranges [2]. The sequential separation of the southern continental blocks since the Mesozoic [3] has led to lineages on multiple post-Gondwanan land fragments forming independent clades. In contrast, oceanic dispersal [1] was often discarded *a priori* as a primary explanation of distribution patterns in the Southern Hemisphere [3]. The idea that seemingly remarkable feats of long-distance dispersal were needed to explain the evolutionary history of many groups of organisms was first postulated by Darwin [4], but the concept has often been considered speculative and difficult to test–“a science of the improbable, the rare, the mysterious and the miraculous” [5]. The apparent poor suitability of many austral groups for oceanic dispersal (e.g. marsupials and ratite birds) appeared to further support vicariance as the more likely biogeographical scenario [3]. Indeed, the idea that vicariance was the key theory to explain the Gondwanan distribution of many southern-temperate groups proved difficult to challenge for many decades [6].

Over the past 20 years, new discoveries and more advanced methods, particularly molecular phylogenetic and dating methods, have brought the dispersal-vicariance debate full-circle. Using the fossil record and/or gene-specific rates of nucleotide evolution, molecular phylogenies with dated nodes now provide new perspectives on the evolutionary history of the flora and fauna of the Southern Hemisphere [6, 7]. Most importantly, molecular divergence dating provides the temporal perspective necessary to test and, where appropriate, reject vicariant biogeographic hypotheses [7]. Calculating the probability of a successful dispersal requires taking into account the number of dispersers, their probability of survival, their likelihood of establishing upon landing, and also the presence of prevailing winds, oceanic currents, hosts, vectors or any other underlying mechanisms that may affect movement and survival (any or all of which may include rafting as a plausible hypothesis) [8]. Recent molecular studies have shown that successful long-distance dispersal events have occurred in many groups of taxa, such as monkeys [9], lemurs [10] and geckos [11], a previously counter-intuitive conclusion without accessible dated molecular phylogenies.

The now well-documented occurrence of long-distance dispersal via rafting in a large range of taxa [8], highlights that trans-oceanic dispersal is not only restricted to organisms capable of flight [12], aerial dispersal (e.g. ballooning spiders [13]) or oceanic buoyancy (e.g. floating seeds [14]). Rafting generally involves large chunks of land and/or vegetation being washed out to sea, with rafting events being implicated in the colonisation of numerous isolated land masses including Australia [3], Madagascar [10,15], South America [7, 9,11], New Zealand [16] and newly formed Darwinian Islands such as the Galapagos islands, Canary Islands and Hawaii [17]. A case in point, and not surprising, is the coastal araneomorph spider genus *Amaurobioides*, which is hypothesised to have undergone several long distance, transoceanic dispersal events, facilitated by rafting [18].Spiders of the infraorder Mygalomorphae are well featured in vicariance biogeography literature (e.g. [19–22]) and more recently in molecular studies of phylogeography and species delimitation [23]. Mygalomorphs are a monophyletic group with a worldwide distribution [24–26]. They have unusually long life cycles, with some species living up to 30 years and requiring 5–8 years to reach reproductive maturity [27]. They are univoltine [28] with females and juveniles leading sedentary lifestyles [29]. Although ballooning of spiderlings has been documented in several genera (e.g. [30–33]) most mygalomorphs do not disperse aerially and are known to be relatively non-vagile, with juveniles often moving only a few metres from the maternal site (e.g. [25,31,34,35]). These life-history traits predispose mygalomorph spiders to geographic isolation through mechanisms such as continental drift, glaciation, orogenic activity and habitat fragmentation, resulting in a large number of taxa that have small geographical distributions [36–38]. It is the poor vagility, sedentary habits and patterns of fine-scale genetic structuring characteristic of many mygalomorph spiders [28] that make this group especially amenable to testing the vicariance paradigm [25].

The Migidae are a family of Mygalomorphae previously assumed to have a vicariant austral distribution. Eleven named genera occur in Africa, Madagascar, New Zealand, New Caledonia, South America and Australia [22,38]. The Australian migid fauna includes four genera: *Migas* L. Koch, 1873 and *Heteromigas* Hogg, 1902 from eastern Australia [39]; *Moggridgea* O. P.-Cambridge, 1904 from Kangaroo Island (KI), South Australia [40]; and *Bertmainius* Harvey *et al*., 2015 from south-western Australia [38]. Although displaying a putatively Gondwanan distribution, a cladistic study based on morphology suggested that the evolutionary history of the family cannot be explained by vicariance alone, with Australia appearing three times in the cladogram [22]. Recent molecular [35,38] and morphological [40] data suggest the only Australian *Moggridgea* species, *Moggridgea rainbowi* (Pulleine, 1919), groups with African *Moggridgea*, where all other congeneric species occur. The existence of an ‘African’ *Moggridgea* lineage in Australia immediately poses a number of tantalising biogeographic questions, and these form the basis of this study.

Here we test three alternative biogeographic hypotheses for the presence of *Moggridgea* in southern Australia, using a dated phylogenetic approach based on a comprehensive multi-gene dataset. The first (null) hypothesis is Gondwanan vicariance, which would be evidenced by a deep and very old divergence date from African congeners, consistent with the age of separation of Africa from the rest of Gondwana. This hypothesis was first suggested by Main [41] to explain the presence of *Moggridgea* (now treated as *Bertmanius*) in Western Australia. An alternative hypothesis (*H*_1_) is a human-mediated introduction from Africa during the European colonisation of KI. This would be evidenced by a recent, extremely shallow (among conspecific) or low divergence date (among sister species) and, equally importantly, by a lack of phylogeographic structure on KI itself. The second alternative hypothesis (*H*_2_) is trans-oceanic dispersal, which would be evidenced by both recent divergence from African species (relative to ancient African vicariance) and by demonstrable phylogeographic structuring among populations on KI. The implications of our results are discussed in regard to their broader impact, as an inability to reject *H*_*2*_ would provide the first dated molecular evidence of long-distance oceanic dispersal in a mygalomorph spider, and would be an invaluable insight into the history and origins of southern hemisphere mygalomorph spider diversity. Our rejection of *H*_*0*_ and *H*_*2*_ provide the first solid evidence for long-distance oceanic dispersal in a mygalomorph spider, and has broader implications for better understanding the history and origins of southern hemisphere mygalomorph spider diversity.

## Methods

### Specimen sampling

Our dataset comprised seven specimens of *M*. *rainbowi* from two populations on KI separated by approximately 80 km (Western River [three specimens] and American River [four specimens]); five exemplar species of *Moggridgea* from South Africa; and seven species of *Bertmainius* from south-western Australia (see Table 1). The American River specimens were excavated from burrows above the high tide mark in May 2013, and initially preserved in 100% ethanol. These specimens were collected under permit number E26155-3 issued by the South Australian Department of Environment, Water and Natural Resources. All *M*. *rainbow* specimens from Western River, *Bertmainius* species from Western Australian and *Moggridgea* specimens from Africa were obtained from archived DNS samples stored in the Australian Biological Tissue Collection, provided with permission from the South Australian Museum. These DNA samples had been previously collected under annual collection permits issued to scientists from the Western or South Australian museums or donated by overseas colleagues. Legs 3 and 4 from the left side of each specimen were then kept in 100% ethanol, while the rest of the body was transferred to 75% to allow for easier manipulation for morphological study. Cytochrome oxidase subunit I (*COI*) and internal transcribed spacer (*ITS*) sequences for *M*. *rainbowi* from Western River (KI), along with the *Moggridgea* species from South Africa and the *Bertmainius* species from Western Australia were taken from [35] and [38]. DNA from these specimens was sequenced for four additional genes: *XPNPEP3*, *28S*, *18S* and *H3* (see below).

**Table 1:** Registration numbers, locality data, and Genbank accession numbers for specimens used in the study.

Species	Registration numbers	Locality	Coordinates	Genbank accession numbers *COI*	Genbank accession numbers *ITS1-ITS2*	Genbank accession numbers *XPNPEP3*	Genbank accession numbers *18S*	Genbank accession numbers 28S	Genbank accession numbers *H3*
SCORPIONES									
*Urodacus planimanus*	T129654	WA: Bedfordale	32°10’05"S, 116°04’06"E	KY295225	-	KY295718	KY294838	KY294961	KY295099
ARANEAE									
*Latrodectus hasseltii*	T129059	WA: Welshpool	31°59’08"S, 115°55’57"E	KY295226	-	KY295719	KY294839	KY294962	KY295100
*Aganippe* sp.	T129362	WA: Serpentine NP	32°22’05"S, 116°00’26"E	KY295228	KY294976	KY295723	KY294841	KY294965	KY295103
*Euoplos* sp.	T129363	WA: Serpentine NP	32°22’05"S, 116°00’26"E	KY598258	KY294983	KY295725	KY294843	KY294970	KY295108
*Cethegus fugax*	T129260	WA: John Forrest NP	31°53’54"S, 116°05’49"E	KY295227	-	KY295722	KY294840	KY294963	KY295101
*Moggridgea rainbowi*	ABTC110307	SA: Western River, Kangaroo Island	35°41’46”S, 136°54’34”E	JF749924	JF749981	MF169599	MF169538	MF169569	MF169628
*Moggridgea rainbowi*	ABTC110308	SA: Western River, Kangaroo Island	35°41’46”S, 136°54’34”E	JF749924	JF749982	MF169600	MF169539	MF169570	MF169629
*Moggridgea rainbowi*	ABTC110309	SA: Western River, Kangaroo Island	35°41’46”S, 136°54’34”E	JF749924	JF749983	MF169601	MF169540	MF169571	
*Moggridgea rainbowi*	SAM NN28257	SA: American River, Kangaroo Island	35°46’35”S, 35°46’35”S	MF169531	MF169535	MF169607	MF169547	MF169577	MF169632
*Moggridgea rainbowi*	SAM NN28345	SA: American River, Kangaroo Island	35°46’36.5"S, 137°46’33"E	MF169532	MF169536	MF169608	MF169548	MF169578	MF169633
*Moggridgea rainbowi*	SAM NN25429	SA: American River, Kangaroo Island	35°46’37”S, 137°46’3"E	MF169530	MF169534	-	MF169546	MF169576	MF169631
*Moggridgea rainbowi*	SAM NN28346.1	SA: American River	35°46’36.5"S, 137°46’33"E	MF169533	MF169537	-	MF169549	MF169579	MF169634
*Moggridgea terrestris*	MY357	South Africa: Eastern Cape Province	33°07’31”S, 26°36’40”E	JF749926	JF749986	MF169602	MF169541	MF169572	-
*Moggridgea rupicoloides*	MY360	South Africa: Eastern Cape Province	33°23’26”S, 26°26’11”E	JF749925	-	MF169603	MF169642	MF169573	-
*Moggridgea intermedia*	MY361	South Africa: Western Cape Province	33°58’13”S, 23°32’20”E	JF749928	JF749984	MF169604	MF169543	-	MF169630
*Moggridgea mordax*	MY371	South Africa: Northern Cape Province Hwy N14	28°01’30”S, 22°39’48”E	JF749929	-	MF169605	MF169544	MF169574	-
*Moggridgea peringueyi*	MY372	South Africa: Northern Cape Province Hwy N14	28°01’30”S, 22°39’48”E	JF749927	JF749985	MF169606	MF169545	MF169575	-
*Bertmainius monachus*	WAM T57952	WA: Stirling Range NP, Talyuberlup	34°24’51”S, 117°57’22”E	JF749911	JF749976	MF169609	MF169550	MF169580	MF169635
*Bertmainius monachus*	WAM T63124	WA: Stirling Range NP, Talyuberlup	34°24’51”S, 117°57’22”E	JF749891	JF749948	MF169624	MF169565	MF169595	MF169645
*Bertmainius monachus*	WAM T63125	WA: Stirling Range NP, Talyuberlup	34°24’51”S, 117°57’22”E	JF749891	JF749972	MF169625	MF169566	MF169596	MF169646
*Bertmainius pandus*	WAM T57954	WA: Stirling Range NP, Toolbrunup	34°23’27”S, 118°03’31”E	JF749912	JF749975	MF169610	MF169551	MF169581	MF169636
*Bertmainius pandus*	WAM T57955	WA: Stirling Range NP, Toolbrunup	34°23’27”S, 118°03’31”E	JF749912	JF749973	MF169611	MF169552	MF169582	MF169637
*Bertmainius colonus*	WAM T57976	WA: Stirling Range NP, Ellen Creek	34°22054”S, 118°17’25”E	JF749906	JF749951	MF169613	MF169554	MF169584	MF169648
*Bertmainius colonus*	WAM T63126	WA: Stirling Range NP, Isongerup Track	34°22’25”S, 118°16’59”E	JF749907	JF749955	MF169626	MF169567	MF169597	MF169647
*Bertmainius colonus*	WAM T58045	WA: Stirling Range NP, Wedge Hill	34°25’12”S, 118°10058”E	JF749909	JF749931	MF169614	MF169555	MF169585	MF169639
*Bertmainius tumidus*	WAM T57961	WA: Porongurup NP	34°40’57”S, 117°50’56”E	JF749892	JF749937	MF169612	MF169553	MF169583	MF169638
*Bertmainius tumidus*	WAM T63106	WA: Waychinicup Nature Reserve	34°54’29”S, 118°15’56”E	JF749923	JF749964	MF169621	MF169562	MF169592	-
*Bertmainius mysticus*	WAM T60315	WA: Keystone State Forest	34°58’59”S, 116°37’53”E,	JF749901	JF749978	MF169615	MF169556	MF169586	MF169640
*Bertmainius mysticus*	WAM T60316	WA: Keystone State Forest	34°58’59”S, 116°37’53”E	JF749915	JF749977	MF169616	MF169557	MF169587	-
*Bertmainius mysticus*	WAM T63096	WA: Walpole–Nornalup NP	35°00’23”S, 116°38’37”E	JF749896	JF749961	MF169617	MF169558	MF169588	-
*Bertmainius tingle*	WAM T63102	WA: Walpole-Nornalup NP, Valley of the Giants	34°58’47”S, 116°52’44”E	JF749921	JF749958	MF169618	MF169559	MF169589	-
*Bertmainius tingle*	WAM T63103	WA: Walpole-Nornalup NP, Valley of the Giants	34°58’47”S, 116°52’44”E	JF749921	JF749956	MF169619	MF169560	MF169590	MF169641
*Bertmainius tingle*	WAM T63104	WA: Walpole-Nornalup NP, Valley of the Giants	34°58’47”S, 116°52’44”E	JF749921	JF749957	MF169620	MF169561	MF169591	MF169642
*Bertmainius opimus*	WAM T63108	WA: S. of Gracetown	33°54’32”S, 115°00’24”E	JF749900	JF749966	MF169622	MF169563	MF169593	MF169643
*Bertmainius opimus*	WAM T63179	WA: Shannon NP	34°42’47”S, 116°21’47”E	JF749920	JF749971	MF169627	MF169568	MF169598	-
*Bertmainius opimus*	WAM T63111	WA: Wellington Mill	33°27’04”S, 115°55’42”E	JF749917	JF749941	MF169623	MF169564	MF169594	MF169644

### Molecular methods

Approximately 3 mm^3^ of muscle tissue was removed from the leg femora for DNA extraction. DNA was extracted using the Gentra DNA extraction PURE-GENE DNA Purification Kit (Gentra Systems, Minnepolis, MN, USA).

A 715 bp fragment of nuclear *18S* rRNA was amplified using the primers 18S_ai (5’-CCTGAGAAACGGCTACCACATC) and 18S_b0.5 (5’-GTTTCAGCTTTGCAACCAT-3’) [42]. PCR was performed under the following conditions: an initial denaturation step of 95°C for 5 mins, followed by 35 cycles of 95°C for 20 s, annealing temperature of 50°C for 35 s, then 72°C for 2 mins, with a final elongation step of 72°C for 10 mins.

An 852 bp fragment of nuclear *28S* rDNA was amplified using the primers 28Sa (5’-GACCCGTCTTGAAACACGGA-3’) and LSUR (5’-GCTACTACCACCAAGATCTGCA-3’) [42]. PCR was performed under the following conditions: an initial denaturation step of 95°C for 5 mins, followed by 35 cycles of 95°C for 20 s, annealing temperature of 50°C for 35 s, then 72°C for 2 mins, with a final elongation step of 72°C for 10 mins.

A 658 bp fragment of mitochondrial *COI* was amplified using the universal *COI* primers LCO1490 (5’-GGTCAACAAATCATAAAGATATTG-3’) and HC02198 (3’-TAAACTTCAGGGTGACCAAAAAATCA-5’) [43]. PCR was performed under the following conditions: an initial denaturation step of 94°C for 5 mins, followed by 34 cycles of 94°C for 45 s, annealing temperature of 48°C for 45 s, then 72°C for 1 min, with a final elongation step of 72°C for 10 mins.

A 738 bp fragment of nuclear Xaa-Pro aminopeptidase 3 (*XPNPEP3*) was amplified using the primers XPNPEP3_f2 (5’-GAAAGAAGATTAAAACTAATGGAAC-3’) and (5’-XPNPEP3_Ar_r1 CCAGCATCCATYAANACCA-3’) [44]. PCR was performed under the following conditions: an initial denaturation step of 95°C for 5 mins, followed by 35 cycles of 95°C for 20 s, annealing temperature dropping from 55°C to 45°C for 35 s, then 72°C for 1 min, with a final elongation step of 72°C for 10 mins.

An 838 bp fragment of nuclear *ITS* rRNA (including *ITS1*, *5*.*8S* rRNA, *ITS2*) was amplified using the primers G923 (5’-CGTAACAAGGTTTCCGTAGGTGA-3’) and G925 (5’AGAGAACTCGCGAATTCCACGG-3’) (see [35]). PCR was performed under the following conditions: an initial denaturation step of 94°C for 9 mins, followed by six cycles of 94°C for 45 s, annealing 68°C for 45 s (-1°C each cycle); 72°C 60 s, then 28 cycles of 94°C for 45 s, annealing 62°C for 45 s, 72°C for 60 s, with a final elongation step of 72°C for 6 min. The enzyme used was AmpliTaq Gold DNA polymerase.

A 327 bp fragment of nuclear histone *H3* was amplified using the primers H3aF (5’-ATGGCTCGTACCAAGCAGACVGC-3’) and H3aR (5’-ATATCCTTRGGCATRATRGTGAC-3’) [45]. PCR was performed under the following conditions: an initial denaturation step of 95^°^C for 5 mins, followed by 35 cycles of 95°C for 20 s, annealing 48°C for 35 s, then 72°C for 2 mins, with a final elongation step of 72°C for 2 mins. The enzyme used was MyTaq DNA polymerase.

The genes *18S*, *28S*, *XPNPEP3* and *H3* were amplified following [44], using MyTaq DNA Polymerase (Bioline, Taunton, MA), in a Bio-Rad T100 Thermal Cycler. For each 25 μL PCR reaction, 2 μL of template DNA, 5 μL of MyTaq buffer, 5 pm of each primer and 0.2 μL of MyTaq DNA polymerase were used. PCR products were visualised on 1.5% agarose gels using standard procedures, and PCR clean-up plus bi-directional sequencing was performed by the Australian Genome Research Facility (AGRF, Nedlands, WA). *COI* and *ITS* were amplified using Eppendorf Amplitaq Gold (Eppendorf, Westbury, NY, USA). For each 25 µL reaction, 2 µL of template DNA, 2.5 µL of PCR Gold Buffer, 3.5 µL of MgCl, 2.0 µL of deoxyribonucleotide triphosphate (dNTP), 10 pm of each primer, and 0.1µL Amplitaq Gold DNA polymerase was used. PCR products were verified by agarose gel electrophoresis (1% agarose), and PCR clean-up plus bi-directional Sanger sequencing was performed by AGRF (Waite Campus, Adelaide, S.A.). Sequences were submitted to GenBank (see Table 1 for accession numbers).

### Phylogenetic analyses

Five non-migid outgroups were sourced from [44]: the scorpion *Urodacus planimanus* Pocock, 1893, the red-back spider *Latrodectus hasseltii* Thorell, 1870, the curtain-web mygalomorph spider *Cethegus fugax* Simon, 1908, and the idiopid trapdoor spiders *Aganippe* sp. O. P.-Cambridge, 1877 and *Euoplos* sp. (Table 1). All newly obtained sequences were edited with reference to chromatograms using Geneious [46]. Forward and reverse sequences were assembled, and the resulting consensus sequences were then aligned using the ‘Geneious Alignment’ function of Geneious. PartitionFinder [47] was used to select the model that best fit each gene, with the protein coding genes being divided into three codon positions. For *COI*, the General Time Reversible (GTR) [48] + gamma (G) [49] model was selected for the first codon position, the Felstein 81 (F81) [50] + invariant (I) I+G for the second codon position, and the Hasegawa, Kishino and Yano (HKY) [51] +I+G for the third. For *ITS1*, *ITS2* and *18S*, the model Kimura 80 (K80) [52] +G was chosen. For *5*.*8S* and *28S*, the GTR+I+G model was chosen. For *H3*, the GTR+I+G model was chosen for codon position one and the K80+G model was chosen for positions two and three. For *XPNPEP3*, the HKY+G model was chosen for all positions.

**Fig 1:**
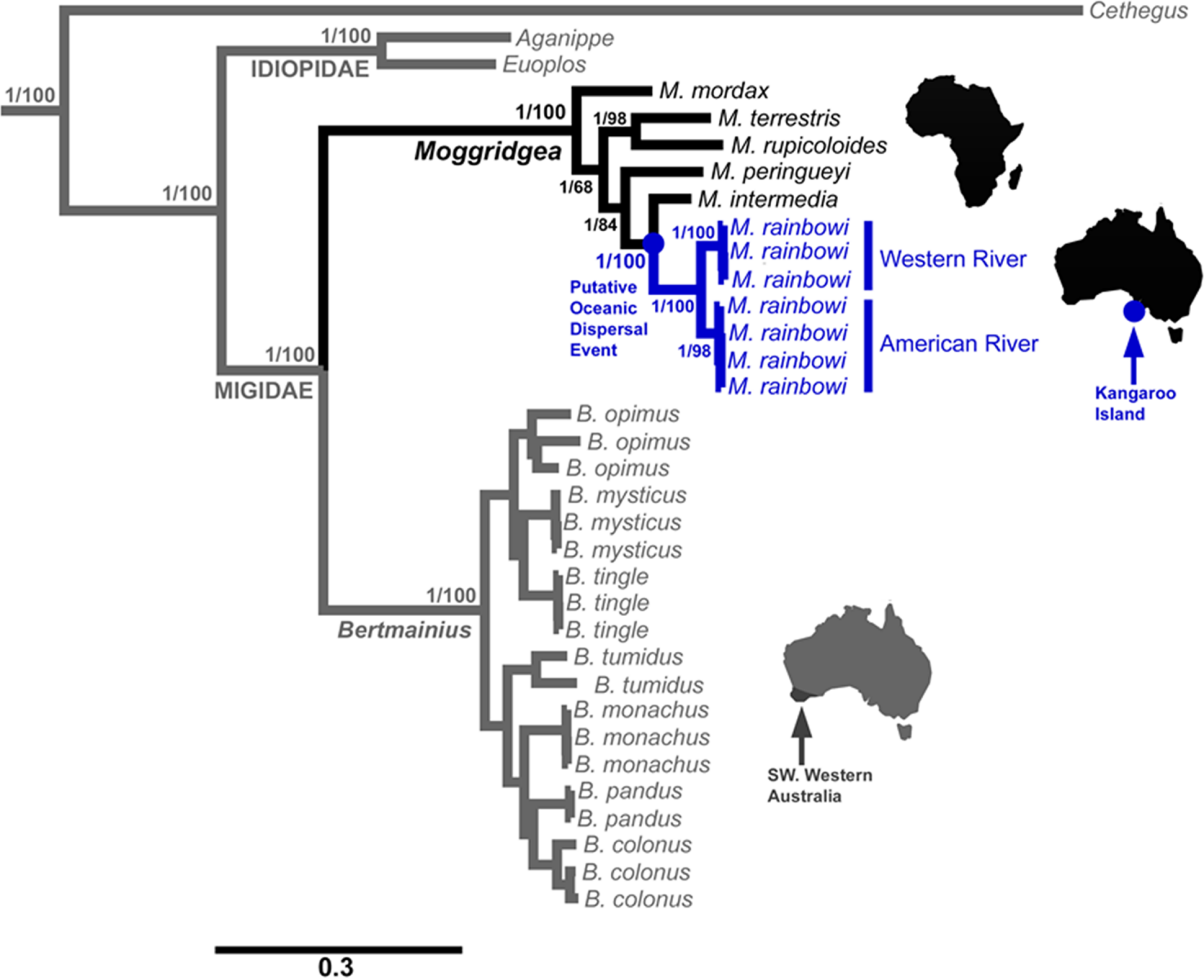
Combined *COI*, *ITS*, *H3*, *18S*, *28S* and *XPNPEP3* tree constructed using MrBayes and mixed models. Numbers on nodes represent posterior probabilities followed by maximum likelihood bootstrap values.

Phylogenetic reconstruction was undertaken using MrBayes 3.2.6 [53] employing the CIPRES Science Gateway [54]. In the Bayesian analysis, each codon position was modelled separately using the models listed above. All parameters were unlinked and the rates were allowed to vary over the partitions. For all reconstructions, two runs with four chains each were run simultaneously for 100 million generations, with every 1,000^th^ tree sampled. A burnin of 1,000, chosen using the program Tracer 1.6 [55], was set for building the maximum clade credibility tree. The resulting tree was viewed using FigTree v1.3.1 [56] (Fig 1). A maximum likelihood analysis was also undertaken using RAxML [57] on the BlackBox server [58] with *COI*, *H3* and *XPNPEP3* partitioned by codons and *ITS1*, *5*.*8S*, *ITS2* and *28S* partitioned individually, with the GTR + G model used for all genes.

### Molecular clock analyses

Divergence dating analysis was performed using BEAST 1.8.0 [59] to determine the time of divergence of *M*. *rainbowi* from its African relatives. The program BEAUti 1.8.0 (part of the BEAST software package) was used to create.xml files to input into BEAST. Given the robustness of phylogenetic analyses placing *M*. *rainbowi* within the African *Moggridgea* clade (see Fig 1), we focused on the *Moggridgea* taxa only for our molecular clock analyses. Exclusion of distantly related taxa, such as *Bertmainius*, avoided potential issues with saturation of the third codon positions of *COI*. This still enabled us to effectively date the nodes of most interest, i.e. the divergence time between the closest African sister to *M*. *rainbowi*, *M*. *intermedia* (see [40]), and the divergence between the two KI *M*. *rainbowi* populations. We included only the specimens for which we had a complete set of sequence data; this allowed us to link the trees and resulted in a single tree for analysis. The gene *H3* had a larger proportion of missing data than the other genes, so was not included in the dating analysis. *28S* was also not included as it could not be sequenced for *M*. *intermedia*, which was found to be the closest relative to *M*. *rainbowi*.

Six separate BEAST analyses were carried out using different clock models, including a strict clock, uncorrelated lognormal clock and exponential relaxed clock, and both the GTR + G + I and HKY nucleotide substitution modes. Each analysis was run for 20 million generations with a burnin of 1 million generations (i.e. 10%), and the program Tracer 1.6 [55] was used to analyse the parameter distributions estimated from BEAST and check for convergence of the chains. Stationarity was checked for, and no evidence of non-stationarity was found in all BEAST runs. As fossil calibrations were unavailable to date nodes of the *Moggridgea* phylogeny, the mean *COI* substitution rate was fixed at 0.02 substitutions per site per million years, based on the estimates of 4% divergence between lineages per million years [by 34] for the trapdoor spider *Aptostichus simus* Chamberlin 1917. Rates for all other genes were estimated. Site models and clock models were unlinked and trees were linked. The tree priors selected for separate analyses were Speciation: Yule Process and Birth-Death Process, as both are suitable for inter-species relationships. Priors on the ucld.mean for each gene were defined as uniform with an initial value of 0.00115, an upper value of 0.0115 and a minimum value of 0.0001. The universal substitution rate estimated for arthropod mtDNA [60] was used to define the upper value. Due to the average slower pace of nuclear genes compared with mitochondrial ones, the initial value was one order of magnitude slower (as per [61]). TreeAnnotator [59] was used to produce a single “target” tree which was then visualised using FigTree v1.3.1 [56].

## Results

### Phylogenetic analysis

A maximum clade credibility tree was generated for the MrBayes analysis of the combined six gene, 4118 character, 36 taxa dataset (Fig 1). This analysis resolved the genera *Moggridgea* and *Bertmainius* as reciprocally monophyletic, with *M*. *rainbowi* from KI clearly embedded within the African *Moggridgea* lineage and sister to *M*. *intermedia* (posterior probability = 1, bootstrap value 100) (Fig 1). Furthermore, *M*. *rainbowi* formed a monophyletic group, but showed phylogeographic structure, with haplotypes reflecting the two geographic locations (Western River and American River). The Maximum Likelihood analysis of the same dataset produced a completely concordant tree (see Dryad digital repository, doi:10.5061/dryad.9cp00).

### Molecular clock analyses

The time to most recent common ancestor (TMRCA) estimate for the African *M*. *intermedia* and KI *M*. *rainbowi*, the TMRCA of the KI Western River and American River populations of *M*. *rainbowi*, and the Posterior Mean and Posterior ESS values for all three clocks with GTR, HKY and PartitionFinder models, using both Speciation: Yule Process and Speciation: Birth-Death Process tree priors are summarised in Table 2. All analyses performed using the GTR+I+G models failed to achieve adequate convergence for many of the parameter estimates (i.e. posterior statistics with effective sample sizes <10 after 20 million generations). Analyses performed using the HKY model had posterior ESS values of >1400 for every clock model used. Combinations of clocks and models gave TMRCA estimates ranging between 2.27 Mya (strict clock, HKY model, Speciation: Yule Process, 95% Highest Posterior Density [HPD] 1.89–2.65) to 16.02 Mya (strict clock, GTR model, Speciation: Yule Process, 95% HPD 8.97–25.60) between the African *M*. *intermedia* and *M*. *rainbowi* from KI. The TMRCA values for the divergence time between the two separate KI populations ranged between 1.10 Mya (Strict clock, HKY model, Speciation: Birth-Death Process, 95% HPD 0.86–1.34) to 6.39 Mya (strict clock, GTR models, Speciation: Yule Process, 3.48–10.23).

**Table 2:** Estimates of time (in millions of years) to most recent common ancestors (TMRCA) and 95% highest posterior density (HPD) intervals for key nodes and posterior mean and effective sample size [ESS] values, generated using BEAST.

Parameters	TMRCA Node 1 (Moggridgea Dispersal) + 95% Highest Posterior Density	TMRCA Node 2 (KI Population Divergence) + 95% Highest Posterior Density	Posterior Mean	Posterior ESS
**Strict Clock, GTR Models, Speciation: Yule Process**	16.02	6.39	-6059.22	20.04
	8.87–25.60	3.48–10.23		
**Strict Clock, HKY Models, Speciation: Yule Process**	2.27	1.10	-6478.87	1449.97
	1.89–2.65	0.87–1.34		
**Strict Clock, GTR Models, Speciation: Birth-Death Process**	15.98	6.35	-5813.92	8.93
	8.63–25.96	3.55–10.37		
**Strict Clock, HKY Models, Speciation: Birth-Death Process**	2.27	1.10	-6259.82	1672.67
	1.91–2.67	0.86–1.34		
**Exponential Clock, GTR Models, Speciation: Yule Process**	10.59	4.06	-6021.34	5.93
	4.01–19.94	1.59–7.66		
**Exponential Clock, HKY Models, Speciation: Yule Process**	3.54	1.75	-6155.69	2310.32
	2.35–4.96	1.17–2.45		
**Exponential Clock, GTR Models, Speciation: Birth-Death Process**	10.49	3.69	-5789.97	7.62
	3.97–19.71	1.60–7.45		
**Exponential Clock, HKY Models, Speciation: Birth-Death Process**	3.54	1.73	-5936.87	1607.35
	2.36–4.92	1.16–2.40		
**Lognormal Clock, GTR Models, Speciation: Yule Process**	15.44	5.96	-6035.79	9.76
	5.36–27.15	1.62–11.13		
**Lognormal Clock, HKY Models, Speciation: Yule Process**	8.48	3.56	-6172.48	1701.01
	3.33–13.97	1.25–6.53		
**Lognormal Clock, GTR Models, Speciation: Birth-Death Process**	15.40	5.77	-5821.38	10.17
	5.41–27.32	1.51–10.63		
**Lognormal Clock, HKY Models, Speciation: Birth-Death Process**	8.48	3.47	-5953.30	1825.83
	3.32–14.12	1.22–6.37		

## Discussion

Our analyses show that *M*. *rainbowi* from KI is unequivocally related to African *Moggridgea*, with KI populations rendering the latter paraphyletic–a result consistent with previous morphological findings [40]. Our six-gene Bayesian analysis is also concordant with previous molecular results [35], with a deep and reciprocally-monophyletic separation between true *Moggridgea* and Australian *Bertmainius*, although the latter study was limited in its taxon and gene sampling and appropriate outgroups to confirm the exact relationships, compared with the current study.

But how did an otherwise African spider lineage end up on KI in southern Australia? To address this question we used two lines of evidence: divergence dating between African and Australian exemplars; and divergence dating between both the KI populations of *Moggridgea*. The (null) hypothesis of Gondwanan vicariance requires a very old divergence date of 110+ Mya between African and Australian *Moggridgea* to be consistent with the vicariant breakup of Gondwana [3]. The inferred split between *M*. *rainbowi* and African *M*. *intermedia* ranged from 2.27–16.02 Mya (Table 2), and the inferred age for the divergence of the two KI populations of *M*. *rainbowi* ranged from 1.10–6.39 Mya. Although there may be considerable uncertainty in the use of a ‘borrowed’ rate for *COI* to estimate divergence times, even if the HPD error margins for our dating estimate were doubled or tripled, it is clear that the dates for these nodes are relatively recent, and not concordant with Africa’s long isolation from the rest of Gondwana. Therefore, vicariance must be rejected as a plausible hypothesis for the presence of *Moggridgea* on KI.

The first of two alternative hypotheses (*H*_1_) is that *Moggridgea* was accidentally introduced from Africa to KI by humans, such as explorers, sealers or European settlers who arrived in 1802 [62]. The sealers came from North America, and settled at what is now American River [63]. If humans brought *Moggridgea* to KI at any time from 1802 onwards (assuming a single introduction), intra-specific phylogeographic structure and genetic divergence equating to 1.10–6.39 Mya of isolation in different regions of KI would be highly unlikely.

While we cannot disprove more than one introduction of the same species, each with divergent mtDNA, to different locations on KI, the probability of two successful dispersal events for the same African species must be very low. Similarly, this hypothesis cannot be rejected on the basis of the divergence of *M*. *rainbowi* from *M*. *intermedia* alone, given our incomplete sampling of African taxa and the possibility of another unknown species in Africa being a closer relative to *M*. *rainbowi*. There is also the possibility of putatively unsampled littoral zone lineages from Africa, which would be more likely to be carried by explorers or oceanic vessels. However, no littoral species are so far known, although concentrated fieldwork would be required to confirm this. More than one introduction also seems highly unlikely due to the significant level of genetic differentiation between *M*. *rainbowi* populations at American River and Western River, which is consistent with *Moggridgea* arriving well before humans colonised the island.

The final hypothesis (*H*_2_) predicts that *Moggridgea* is present in Australia due to long-distance dispersal from southern Africa. This proposition, which best fits the estimated divergence date of 2.27–16.02 Mya, cannot be rejected given current morphological and molecular evidence, and is our preferred explanation for the data. Long-distance dispersal of 10,000 km may be improbable for a sedentary trapdoor spider such as *Moggridgea*, but oceanic dispersal is not unprecedented for this genus, at least over shorter distances. Most species occur on mainland Africa, but three species are known from offshore islands. These include *M*. *occidua* (Simon, 1907) from Príncipe, *M*. *nesiota* Griswold, 1987 from Comoros, and *M*. *socotra* Griswold, 1987 from Socotra [64,40]. Príncipe and Socotra are both continental fragments of mainland Africa, and therefore their fauna may have originated by vicariance and not dispersal. However, the Comoros are volcanic in origin and were formed between 0.1 and 7.7 Mya [65]. *Moggridgea nesiota* Griswold 1987b is found on the island of Moheli, which was formed 5.5 Mya, suggesting that the presence of this species there can only be explained by dispersal from mainland Africa (approximately 340 km away). Although only a small fraction of the distance between the south-western Cape and KI, this distribution does suggest that *Moggridgea* is capable of oceanic dispersal, most likely facilitated by rafting given their burrow-dwelling existence. Colonisation by individuals who have arrived via rafting will inevitably occur in the littoral zone [66] which is consistent with the habitat of *M*. *rainbowi* at American River, where their burrows have only been found in vertical banks just above the high tide mark [40]. This habitat also provides further evidence of an unusual, possibly high degree of salt tolerance.

While this study represents the first robust evidence of long-distance trans-oceanic dispersal in a mygalomorph spider, oceanic dispersal at a smaller scale (e.g. as for *M*. *nesiota*) can be inferred for several other mygalomorphs. This is especially so for those species that occur on newly formed islands of volcanic origin (e.g. Galapagos Islands and Hawaii), and those that were once connected to a continental landmass, such as the Seychelles, the latter of which are part of the granitic Mascarene Plateau which broke off from the Indian Plate about 66 Mya) [17]. While some mygalomorphs found on non-continental islands are capable of ballooning (e.g. *Ummidia* Thorell, 1878 which is present on the volcanic island Saint Vincent in the Caribbean [67]; and *Conothele* Thorell, 1878 which occurs on some Pacific Islands and the Seychelles [68,69]), there are also other mygalomorphs that cannot disperse this way, and yet are present on young, isolated landforms. The barychelid *Nihoa hawaiiensis* (Raven, 1988) [70] occurs on the Leeward Islands [71] which form part of the Society islands and has a very recent age progression of 1–4.5 Mya [72]. Species of a second barychelid genus, *Idioctis* L. Koch, 1874 inhabit numerous islands (i.e. Fiji, Western Samoa, Madagascar, the Seychelles, Christmas Island, and Caroline and Marshall Islands), as well as the intertidal or littoral zones of northern Australia, New Caledonia and the Solomon Islands [71]. Their habitat and distribution suggests oceanic dispersal may be the most plausible hypothesis to explain their distribution patterns [70]. A third barychelid genus, *Sason* Simon, 1887, occurs in the Seychelles, the Andaman and Mariana Islands, southern India, Ceylon, northern Australia and New Guinea [73]. Their arboreal nests may render them more amenable to oceanic travel; if an entire log or tree was dislodged and became oceanic flotsam, survival of a trans-oceanic journey may have been possible [19,20]. However, while the evidence supporting these hypothesised oceanic dispersals is compelling, none are yet supported by dated molecular phylogenies.

The direction of dispersal events can help draw conclusions about the origin of taxa. For example, taxa in Hawaii which rely on wind dispersal, such as birds and spiders, come primarily from the east [66], as predicted by storm patterns. However, taxa that disperse via rafting come mostly from the west, as predicted by oceanic currents. For dispersal via rafting, these currents may assist in the movement of buoyant objects, such as seed pods, over long distances [14]. Similarly, these currents could also be a driving force in the movement of a large vegetation rafts and other debris from Africa to Australia.

The origin of much of Australia’s mygalomorph fauna has been attributed to invasion from the north and south of the continent [74], however the potential mechanisms of dispersal are not known [see 73]. The main difficulties with trans-oceanic dispersal for mygalomorphs have been discussed for ground spiders [75], and include prolonged exposure to desiccating atmosphere, lack of non-saline water, and the extremely small probability of juveniles settling in a suitable habitat, maturing, and mating [20]. However, dispersal via rafting cannot be ruled out for migids [22]. There are a number of other factors worth considering which may lead trapdoor spiders to be suited to oceanic dispersal, such as their low metabolic rate [76]. The use of silk-lined burrows with a snugly fitting trapdoor provides a relatively stable microhabitat, enabling trapdoor spiders to regulate temperature and humidity [77]. If a rafting event was facilitated by the movement of a large mass of earth or whole trees, it is plausible that spider burrows may remain intact for long periods. Nest building, defined as thickened silk placed in a pre-existing niche or cavity (requiring minimal excavation) has been well documented in African *Moggridgea*, and is more prevalent than true burrow building [64]. This method allows spiders to colonise arboreal habitats, which may aid their dispersal. Dispersal by a gravid female capable of producing numerous juveniles, would enhance the chance of a successful dispersal event and subsequent mating [20]. In addition, the ability of mygalomorph spiders to resist drowning and use stored oxygen is a critical survival tactic in terrestrial environments when burrows are temporarily flooded [78], and the same is likely to be true on oceanic rafts. While there is no doubt that large expanses of seawater pose a significant challenge to oceanic dispersal, they are clearly not insurmountable barriers, given enough time. With their low food intake requirements, protective burrows, and ability to ‘hold their breath’, small trapdoor spiders may be even better equipped for dispersal than previously realised.
